# Habitual physical activity in patients born with oesophageal atresia: a multicenter cross-sectional study and comparison to a healthy reference cohort matched for gender and age

**DOI:** 10.1007/s00431-023-04923-3

**Published:** 2023-03-28

**Authors:** Tatjana Tamara König, Maria-Luisa Frankenbach, Emilio Gianicolo, Anne-Sophie Holler, Christina Oetzmann von Sochaczewski, Lucas Wessel, Anke Widenmann, Leon Klos, Simon Kolb, Jannos Siaplaouras, Claudia Niessner

**Affiliations:** 1 Department of Pediatric Surgery, Universitätsmedizin, Johannes Gutenberg-University Mainz, Langenbeckstr. 1, Mainz, 55131 Germany; 2 Institute of Medical Biostatistics, Epidemiology and Informatics (IMBEI), Universitätsmedizin, Johannes Gutenberg-University Mainz, Mainz, Germany; 3 Institute of Clinical Physiology, National Research Council, Lecce, Italy; 4 Department of Pediatric Surgery, Dr. von Hauner Children’s Hospital, University Hospital, LMU Munich, Munich, Germany; 5 Sektion Kinderchirurgie, Klinik und Poliklinik Für Allgemein-, Viszeral-, Thorax- Und Gefäßchirurgie, Universitätsklinikum Bonn, Bonn, Germany; 6 Pediatric Surgery, University Medical Centre Mannheim, Mannheim, Germany; 7 Patient Organisation for Esophageal Diseases KEKS e.V., Stuttgart, Germany; 8 Institute of Sports and Sports Science (IfSS), Karlsruhe Institute of Technology, Karlsruhe, Germany; 9 University of Applied Sciences Fulda, Health Sciences, Fulda, Germany

**Keywords:** Rare diseases, Tracheoesophageal fistula, Sports, Physical education, Children, Adolescents

## Abstract

Oesophageal atresia (EA) is associated with life-long gastrointestinal and respiratory morbidity and other associated malformations. The aim of this study is to compare physical activity (PA) levels of children and adolescents with and without EA. A validated questionnaire (MoMo-PAQ) was used to evaluate PA in EA patients EA (4–17 years), who were randomly matched for gender and age (1:5) with a representative sample of the Motorik-Modul Longitudinal Study (*n* = 6233). Sports activity per week (sports index) and minutes of moderate to vigorous physical activity per week (MVPA minutes) were calculated. Correlations between PA and medical factors were analysed. In total, 104 patients and 520 controls were included. Children with EA were significantly less active at higher intensities (mean MPVA minutes 462; 95% confidence interval (CI): 370–554) compared to controls (626; 95% CI: 576–676), although there was no statistically significant difference in the sports index (187; 95% CI: 156–220 versus 220; 95% CI: 203–237). A lower mean weight-for-age and height-for-age, additional urogenital (*r* =  − 0.20, *p* = 0.04) or anorectal malformation (*r* =  − 0.24, *p* = 0.01) were associated with fewer MVPA minutes. For other medical factors (prematurity, type of repair, congenital heart disease, skeletal malformation or symptom load), no statistically significant association with PA was found.

*  Conclusion*: EA patients participated in PA at a similar level but lower intensities compared to the reference cohort. PA in EA patients was largely independent of medical factors.

*Trial registration*: German Clinical Trials Register (ID: DRKS00025276) on September 6, 2021.

**Table:** 

**What is Known:**
• *Oesophageal atresia is associated with a low body weight and height, delayed development of motor skills and impaired lung function and exercise capacity*.
**What is New:**
• *Patients with oesophageal atresia have a similar amount of sports activity per week but engage significantly less in moderate to vigorous physical activities compared to peers.*
• *Physical activity was associated with weight-for-age and height-for-age, but largely independent of symptom load and other medical factors.*

**Supplementary Information:**

The online version contains supplementary material available at 10.1007/s00431-023-04923-3.

## Introduction

Oesophageal atresia (EA) is a rare malformation that is frequently combined with tracheal malformations, such as tracheoesophageal fistula and tracheomalacia [1]. Even after successful repair, patients suffer from long-term gastrointestinal [2], respiratory [3] and developmental problems [4]. Furthermore, EA is associated with congenital heart disease and other congenital malformations (e.g. VACTERL association) and can be complicated further by prematurity and low birth weight [5]. After repair, complications may lead to frequent hospitalization, especially during the first year of life [5]. In cases of long-gap EA, primary anastomosis in the newborn period is impossible, and patients may be hospitalized for months before definitive repair is achieved [6].

Physical activity (PA) is an important factor for children’s physical and mental health [8]. The World Health Organization recommends a mean of 60 min of moderate to vigorous physical activity (MPVA) daily for children and adolescents, including at least 3 days of vigorous activities that increase muscle strength [9]. This is a recent update of the previous version from 2010 recommending 60-min MVPA every single day [10]. These recommendations also apply for children and adolescents with chronic disease or disability. In these cases, it is recommended to set individual sustainable goals [9]. In patients born with EA, delayed development of motor skills [4, 11] and impaired exercise capacity [12, 13] have been described. Furthermore, longer cumulative anaesthesia duration was associated with gross motor problems [7]. These problems might have a negative impact on the level of physical activity, while sports participation was associated with better motor skills in patients with EA [7].

To the best of our knowledge, there is no study investigating physical and sports activity behaviour of children born with EA. The aim of this study is to describe physical activity and influencing factors in children born with EA compared to a representative reference cohort.

## Materials and methods

### Data

In this cross-sectional multicenter study, 251 families of EA patients between aged 4–17 years were personally contacted by the participating center (September 2021–July 2022). An open invitation was issued to all members of the patient organization KEKS e.V. (492 members 4–17 years). As reference cohort, data from the Motorik-Modul Longitudinal Study (MoMo, 2009–2021, *n* = 6233), was used. The MoMo Study is a nationwide study on physical activity and physical fitness in children and adolescents living in Germany. It is an in-depth study of the German Health Interview and Examination Survey for Children and Adolescents (KiGGS) [14]. In order to ensure a diverse sample of children and adolescents and to maximize representativeness, a nationwide, stratified, multi-stage sample subject recruitment was carried out within the KiGGS in two steps [15], considering the level of urbanization and the geographic distribution before randomly selecting age-stratified youth from the official registers of residents. Therefore, the MoMo sample can be regarded as a reflection of the general population of children and adolescents in Germany [14]. For each EA patient, five individuals of the same sex and matching age were randomly selected from the MoMo study participants.

### Questionnaire

For EA patients, the questionnaire included a condition-specific medical history. For patients and controls, questions on biometric data, social background and the duration, frequency and intensity of habitual PA per week were queried according to the standardized and validated MoMo-Physical Activity Questionnaire (MoMo-PAQ). The questionnaire consists of 28 items, including questions on sports activity (curricular and extracurricular school sports, sports club, leisure sports) and everyday activities (chores, outdoor play, active transportation) and has been validated by correlation with accelerometer data [16]. The intensity of PA was classified according to a subjective scale: light “no sweating or heavy breathing”, moderate “a little sweating and heavy breathing” or vigorous “a lot of sweating and heavy breathing” [17].

The sports index is the sum of sports activities per week in kindergarten or school, organized or unorganized sports. Seasons, weekends and school holidays were considered. Further, the total amount of MVPA per week was calculated as a sum of sports and everyday activities, including outside play, active transportation and chores around the house and garden with a minimum of “a little sweating and heavy breathing”. The number of days with at least 60 min of MPVA was analysed separately in order to determine if participants fulfilled the WHO recommendation of 2010 [10].

Participants with missing sex, age, EA type or incomplete MoMo-PAQ were excluded. Default setting of questions on patient history was “unknown”. Missing answers were therefore rated and reported as such. These participants were excluded from analysis with regard to the unknown variable.

### Statistical analysis

Z-scores of the weight- and height-for-age distributions were determined according to Kromeyer-Hauschild. Means and associated 95% confidence intervals (95% CI) were calculated for the sports index and MVPA minutes. Non-overlapping 95% CI was considered statistically significant. χ^2^ test (two categories) or Mann–Whitney U test (multiple categories) were performed for categorical data. Spearman rank correlation coefficient or bivariate linear regression (Pearson’s coefficient) was calculated for continuous variables as appropriate. *P* < 0.05 was considered statistically significant. The analysis was performed using IBM SPSS Statistics, Version 27.0.1.0.

## Results

The overall response rate was 26% (invited families 38%, patient organization 20%). In total, 104 patients and 520 controls (55% male, 45% female) from the reference cohort were included (Supplement 1).

### Oesophageal atresia group characteristics

The vast majority (74%) of patients was underweight according to the International Obesity Task Force classification. The mean weight-for-age was *z* =  − 0.7 (95% CI: − 1.1; − 0.4, missing *n* = 4), and the mean height-for-age was *z* =  − 0.2 (95% CI: − 0.6; 0.1, missing *n* = 2, (Table 1). In bivariate linear regression, there was a stronger effect of biometric factors on the mean MVPA minutes than the sports index, which was statistically significant for former (at the age of 4 years) and current weight-for-age and height-for-age and MVPA minutes (Table 1).

**Table 1:** Former (age 4 years) and current mean weight-for-age, height-for-age and BMI-for-age and bivariate linear regression with sports index and MVPA minutes

		**Linear regression sports index**		**Linear regression MVPA minutes**	
	**Mean z-score (95% CI)**	**Pearson r**	***p***	**Pearson r**	***p***
Weight-for-age at 4 years	−1.1 (− 1.4; − 0.8)	0.01	0.36	0.29	0.008*
Height-for-age at 4 years	−0.9 (− 1.2; − 0.6)	0.08	0.48	0.25	0.023*
BMI-for-age at 4 years	−0.74 (− 0.99; − 0.49)	0.09	0.44	0.20	0.072
Current weight-for-age	−0.7 (− 1.1; − 0.4)	0.20	0.047*	0.27	0.006*
Current height-for-age	−0.2 (− 0.6; 0.1)	0.19	0.066	0.29	0.003*
Current BMI-for-age	−0.78 (− 1.1; − 0.5)	0.14	0.16	0.14	0.167

*BMI* body mass index, *MVPA* moderate to vigorous physical activity, *95% CI* 95% confidence interval

*Statistically significant relationship (*p* < 0.05)

Patient characteristics are summarized in Table 2. No relevant association with of any of the analysed medical factors on the sports index could be shown. However, weak but a statistically significant association with MVPA minutes was shown for the type of EA (*r* = 0.20, *p* = 0.04), additional urogenital malformation (*r* =  − 0.20, *p* = 0.04) and additional anorectal malformation (*r* =  − 0.24, *p* = 0.01). There was no association of PA with prematurity, primary versus secondary anastomosis, open versus minimally invasive repair, number of procedures under general anaesthesia or congenital heart disease with or without current treatment (Supplement 2).

**Table 2:** Patient characteristics

		***n* (%)**
Oesophageal atresia classification	Gross type A	8 (7.7%)
	Gross type B	7 (7.6%)
	Gross type C	87 (84%)
	Gross type D	0
	Gross type E	2 (1.9%)
Premature birth		53 (51%)
Timing of anastomosis	Primary anastomosis	86 (83%)
	Delayed anastomosis	13 (13%)
	Unknown	2 (1.9%)
Surgical procedures	Open repair	63 (61%)
	Minimally invasive repair	29 (28%)
	Both open and minimally invasive repair	2 (1.9%)
	Unknown	10 (9.6%)
	Gastrostomy	27 (26%)
	Unknown	13 (13%)
	Fundoplication	15 (14%)
	Unknown	6 (5.8%)
	Oesophageal dilatations	52 (50%)
Number of procedures under general anaesthesia	1–5	57 (55%)
	6–10	15 (14%)
	11–15	8 (7.7%)
	> 15	20 (19%)
	Unknown	4 (3.8%)
Associated malformation	Congenital heart disease without treatment	28 (27%)
	Congenital heart disease with current treatment	14 (14%)
	Skeletal malformation	26 (25%)
	Urogenital malformation	26 (25%)
	Anorectal malformation	13 (13%)
	Other	33 (22%)
Symptoms	Current symptoms at rest	29 (28%)
	GERD at rest	26 (25%)
	Respiratory symptoms at rest	9 (8.7%)
	Current symptoms during exercise	27 (26%)
	GERD during exercise	6 (5.8%)
	Respiratory symptoms during exercise	21 (20%)

*GERD* gastroesophageal reflux disease

Overall, 28% of EA patients currently had symptoms at rest: 25% suffered from gastroesophageal reflux and 8.7% from respiratory symptoms. During exercise, respiratory symptoms were predominant (20% of patients), while only 5.8% suffered from gastroesophageal reflux symptoms (Table 2). There was no statistically significant correlation between symptoms and PA (sports index/MVPA minutes, Supplement 2).

Out of the 69 school children, 66 (96%) participated in regular physical education in school, compared to 95% in the reference group. Restrictions for physical activity were given to ten patients by their attending doctors (Supplement 3). These patients had multiple co-morbidities, and restrictions were mostly unrelated to EA. Only one patient had restrictions due to severe gastroesophageal reflux. One patient had a full school sports exemption, and one was exempt from being graded in physical education (Supplement 3).

There was a mild but statistically significant positive effect on the sports index, when participants’ siblings were member of a sports club (*r* = 0.27, *p* = 0.02). There was no statistically significant correlation with either sports index or MVPA minutes for patients whose mother or father were physically active or member of a sports club (Supplement 4).

### Comparison to reference cohort

**Fig. 1:**
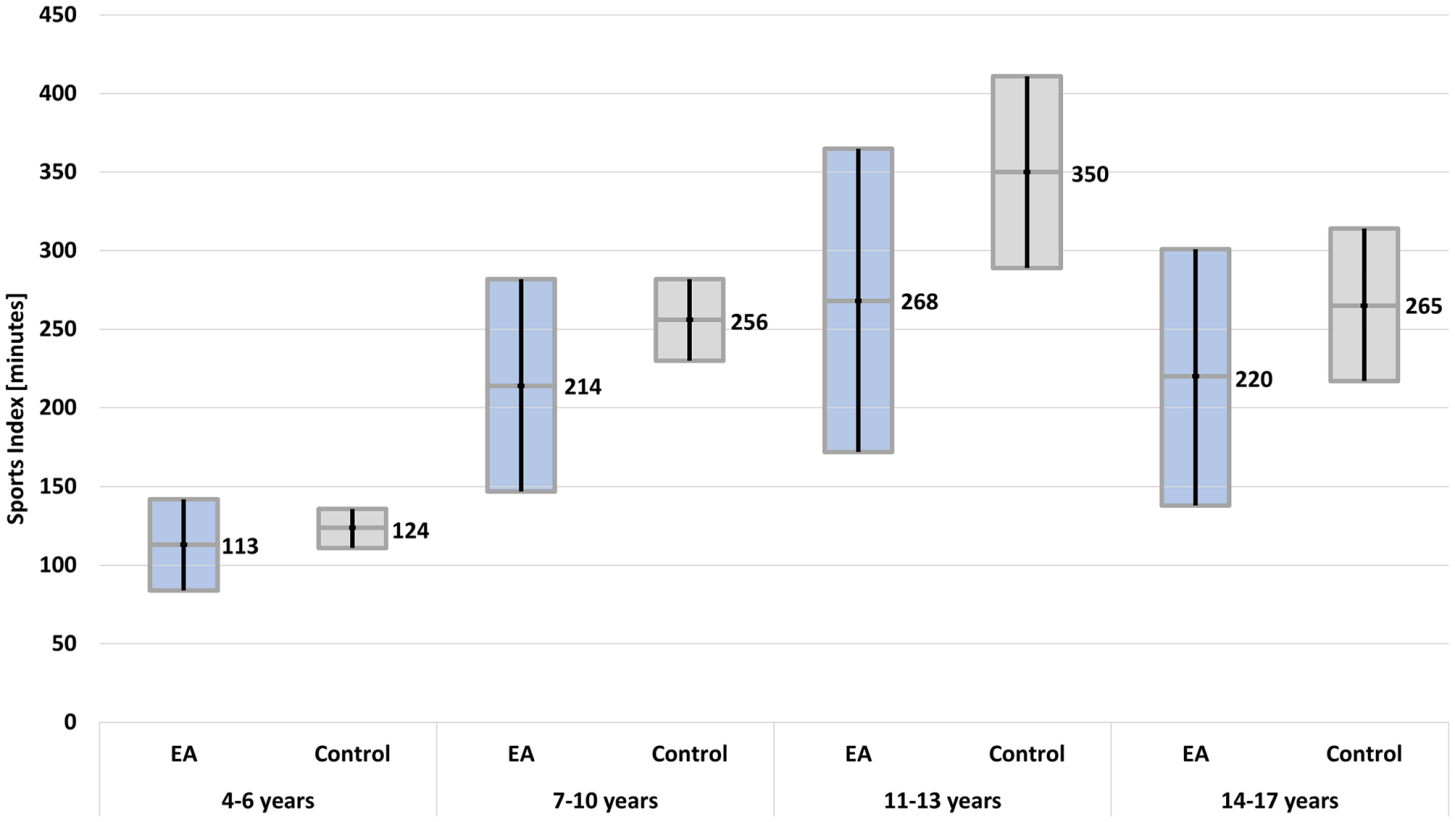
Mean sports index in minutes per week with 95% confidence interval according to age in patients compared to the control group. EA, oesophageal atresia

**Fig. 2:**
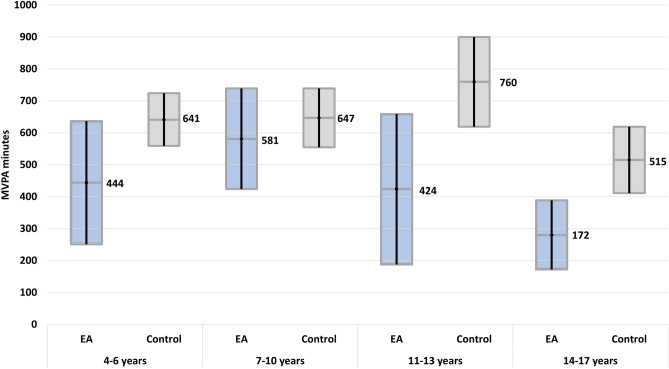
Mean MVPA in minutes per week with 95% confidence interval according to age in patients compared to the control group. EA, oesophageal atresia; MVPA, moderate to vigorous physical activity

Overall, the majority of EA patients was less active compared to the reference cohort (Table 3). There was a trend towards a lower sports index in EA patients for both genders that was not statistically significant (Table 3). EA patients, especially girls, had a significantly lower mean amount of MVPA minutes per week (462, 95% CI: 370–554) compared to controls (626, 95% CI: 576–676, Table 3). Overall, there was a wide range in the sports index and MVPA minutes between individuals, as indicated by large confidence intervals (Table 3, Figs. 1 and 2). The mean number of days with at least 60-min MVPA was the same in both groups (Table 3). A relevantly higher percentage (27%) of EA patients fulfilled the WHO recommendation of 2010 in the EA group compared to controls (18%, *p* = 0.04, Table 3).

**Table 3:** Physical activity of patients compared to controls

	**Patients**	**Controls**	
*n* total	104	520	
*n* male	57	285	
*n* female	47	235	
Mean age (95% CI)	8.6 (7.9–9.4)	8.4 (8.0–8.7)	
Mean sports index (95% CI)	187 (156–220)	220 (203–237)	
Minimum sports index (minimum–maximum)	0–917	0–1727	
Mean sports index (95% CI) males	203 (155–251)	228 (204–253)	
Mean sports index (95% CI) females	169 (127–210)	209 (187–232)	
Mean MVPA minutes/week (95% CI)	462 (370–554)*	626 (576–676)*	
MVPA minutes/week (minimum–maximum)	0.0–2887	1.4–4286	
Mean MVPA minutes/week (95% CI) males	576 (432–719)	657 (588–725)	
Mean MVPA minutes/week (95% CI) females	324 (220–429)*	636 (559–713)*	
Mean number of active days (> 60 min MVPA/day)	4.4 (4.0–4.8)	4.2 (4.1–4.4)	
Participants fulfilling WHO recommendations (2010), *n* (%)	28 (27%)	94 (18%)	*p* = 0.04**

*MVPA* moderate to vigorous physical activity, *WHO* World Health Organization, *95% CI* 95% confidence interval

*Statistically significant difference with non-overlapping 95% CI; **Statistically significant difference (*p* < 0.05, χ^2^ test)

The sports index was mainly characterized by moderate PA (EA 74%, controls 75% of patients, EA mean 144 min, control mean 177 min, Supplement 5). More EA patients (31%) participated with a low intensity compared to controls (21%). During school sports, 6.7% of EA patients and 21% of controls participated with vigorous intensity. In the analysis according to age group, participants of the control group had a higher mean sports index (Fig. 1) and a higher number of mean number of MVPA minutes per week (Fig. 2) compared to EA patients. However, confidence intervals were wide, and the difference was not statistically significant. In both groups, the highest sports index was observed in the age group of 11–13-year-olds (Fig. 1). MVPA minutes peaked at 11–13 years in the control group and at 7–10 years in the patient group (Fig. 2).

A comparable percentage of participants was a member in a sports club in both groups (EA 58%, control 62%, *p* = 0.36). A higher fraction of EA patients participated with light intensity (19% vs. 9.0%) However, significantly more children in the control group participated in sports competitions (EA 25%, control 40%, *p* < 0.01). PA of the participant’s mothers, fathers and siblings was similar in patients and controls (Supplement 6). Notably, there was a statistically significant difference in the percentage of only children in EA patients (25%) compared to controls (12%, *p* < 0.001). Most EA patients (55%) did not have to perform chores around the house or garden, while most controls (88%) did. Most EA patients walked or cycled at low intensity (80%), while most controls walked or cycled with moderate intensity (84%).

## Discussion

To the best of our knowledge, the present study is the first study investigating children and adolescents with EA for their physical and sports activity behaviour. Our study shows a statistically significant lower amount of total MVPA minutes per week in children with oesophageal atresia compared to controls, which was more pronounced in girls. For sports activity, we could show a trend that children with EA were less active and more frequently participated with lower intensities compared to controls and were significantly less likely to participate in sports competitions.

As expected, the response rate was roughly two times higher in the families that were invited personally. According to a recent meta-analysis of 1071 comparable studies, response rates most frequently ranged between 20 and 40%, as it does in our study [18].

Regular physical activity improves physical fitness, prosocial behaviour and healthy sleep [19] and is crucial for cardiometabolic health, bone health, mental health and cognitive outcome [8, 19]. Physical education in school is one of the most important tools to assure a minimum of PA independent of the social background. Additional long-term participation in extracurricular sports and sports clubs leads to a relevant increase in sports competence, endurance and increase of MVPA minutes per week, especially in children and adolescents, who participate in sports competitions [20]. For children with chronic conditions, these activity-induced benefits are especially valuable to prevent further health deterioration. In contrast to the general population, a lower weight-for-age and height-for-age in EA patients were associated with a lower amount of MVPA. Data on body composition was not considered in our study, but a low muscle mass and function (sarcopenia) and relatively high fat mass (obese sarcopenia) are a common finding in children and adolescents with severe chronic conditions in spite of a low or normal BMI [21]. Sarcopenia has adverse effects on growth, neurocognitive development, immune function and health-related quality of life [22]. For children with EA, reduced muscle strength in a standardized motor test has been reported previously [11]. Therefore, the lower PA in children with lower weight-for-age might be associated with sarcopenia. More research is needed on this subject.

The level of PA in EA patients was widely independent of medical factors, such as co-morbidity, minimally invasive versus open repair, total number of procedures under general anaesthesia and current symptoms. EA patients participated in physical education in school at the same rate as the reference cohort. Sports restrictions issued by physicians were largely unrelated to EA. Associated malformations, especially congenital heart disease (CHD), are often believed to lead to sports restrictions. However, children with CHD are wrongly exempt from school sports by their doctors more often than strictly necessary [23]. In our cross-sectional cohort, additional anorectal or urogenital malformation was associated with fewer MVPA minutes per week, indicating that a combination of relevant malformations, or even VACTERL, might be problematic for PA. Contrary to our expectations, neither CHD nor skeletal malformation had a statistically significant association with a lower sports index or MVPA minutes, even though present in a relevant percentage of included patients.

According to a recent meta-analysis, thoracoscopic repair has a lower risk for musculoskeletal complications compared to thoracotomy [24], especially when compared to conventional non-muscle sparing lateral thoracotomy with serratus anterior muscle division [25]. In our cohort, one patient reported scapula winging, but no patient reported musculoskeletal complications causing a limitation to sports participation, even though the majority of patients had an open repair. This finding supports the notion that many subclinical sequelae were not of clinical relevance in the context of PA.

Respiratory symptoms during PA are one of the items in the condition-specific questionnaire for health-related quality of life EA-QOL [26], indicating the relevance of this issue on the patients’ everyday life. In our cohort, EA-related symptoms were present in more than 25% of patients. The main complaint at rest was associated with gastroesophageal reflux, while respiratory symptoms were more prevalent during PA, representing the main subjective limiting factor for exercise tolerance. Most patients complained of dyspnea linked to tracheomalacia during exertion. For adolescents with EA, a significantly lower vital capacity and a high prevalence of restrictive ventilatory disorders have been described [12, 27], which might be rooted in recurrent pulmonary infections in early childhood [3]. Surprisingly, even respiratory symptoms during PA were not associated with a statistically significant decrease of sports index or MVPA minutes in our cohort. One possible explanation is that intensity was linked to the subjective amount of heavy breathing in our standardized questionnaire. In children with respiratory problems, “heavy breathing” may occur at lower intensities compared to other patients. Therefore, further evaluation with more objective measures, such as heart rate, might be warranted for the evaluation of PA intensity in patients with respiratory problems.

The social background plays an important role in sports participation and physical activity [20, 28]. In a cohort of children and adolescents with CHD, physical activity of the father was a predictor for PA of the child [23]. In our cohort, there was a mild association between the activity of patients and their siblings, even though a high percentage of children in the EA group were only children.

According to the WHO recommendation for children and adolescents with chronic health conditions, regular PA with suitable intensity and duration according to the individual’s level is the key to profit from PA [19]. Participants of the control group had a slightly higher mean sports index but significantly more MVPA minutes per week and performed activities at higher intensities compared to EA patients. On the other hand, more EA patients (27%) fulfilled the WHO recommendation of 60 min daily MVPA compared to the reference cohort (19%) or the general population (15% [29]).

Physical activity typically changes with gender and age and depends on a number of developmental and social factors and the physical self-concept [28]. It typically decreases at the time of the transition from primary to secondary school [29], coinciding with the onset of pubescence. Accordingly, sports index and MVPA minutes changed with gender and age in both groups in our study: the sports index peaked between the ages of 11 to 13 years in both groups. MVPA minutes peaked earlier in EA patients (at 7–10 years) than in the control group (at 11–13 years). As described in the literature for the general population [29], girls were less active than boys in both the EA and the control group. In addition, girls with EA were substantially less active than female controls, while there was no statistically significant difference in boys compared to peers, even though males have a higher morbidity and even mortality associated with EA [30]. Since PA was widely independent of morbidity associated with EA, the same rules apply as they do for all children and adolescents: interventions to improve PA should target the social environment and the individuals’ physical self-concept [28].

### Limitations

Data on the medical history was provided by the participants, not medical professionals and phrased accordingly. Further, selection bias in the EA group in favour of families, who are interested in physical activity and sports, cannot be ruled out. Notably, the patients’ families were equally active in comparison to the control group (Table 3), suggesting a comparable social background with regard to PA.

## Conclusions

Children and adolescents with oesophageal atresia had a similar level of sports activity compared to the reference cohort and participated in regular physical education in school. In accordance with the WHO recommendations for children and adolescents with chronic health conditions, EA patients tended to engage at lower intensities, with equal or even higher frequencies compared to controls. The mean weekly duration of activities with moderate to vigorous intensity in EA children was significantly lower, and they were less likely to participate in sports competitions. Especially adolescent girls with EA were significantly less active compared to their peers. A lower weight-for-age and height-for-age were associated with a lower level of PA. For other medical factors, no relevant association could be shown. During medical follow-up, the question of physical activity should be addressed. Medical limitations associated with EA are rare but should be tackled in order to promote physical activity, if present. Otherwise, PA should be promoted in the same way as for the general population.

## Supplementary Information

Below is the link to the electronic supplementary material.

Supplementary file1 (PDF 166 KB)

Supplementary file2 (PDF 115 KB)

Supplementary file3 (PDF 124 KB)

Supplementary file4 (PDF 107 KB)

Supplementary file5 (PDF 118 KB)

Supplementary file6 (PDF 111 KB)

## Data Availability

Data concerning esophageal atresia patients generated or analysed during this study are included in this published article and its supplementary information files. Data of the control group that support the findings of this study are available from the MoMo-Study Group (momo@ifss.kit.edu), but restrictions apply to the availability of these data,which were used under license for the current study, and therefore are not publicly available. Data are however available from the authors upon reasonable request and with permission of MoMo project management and leadership.

## Abbreviations

BMI: Body-mass-index
CHD: Congenital heart disease
EA: Oesophageal atresia
MVPA: Moderate to vigorous physical activity
PA: Physical activity
WHO: World Health Organization
