# Supplementary material for: Habitual physical activity in patients born with oesophageal atresia: a multicenter cross-sectional study and comparison to a healthy reference cohort matched for gender and age
Source: Eur J Pediatr. 2023 Mar 28;182(6):2655–63. doi: 10.1007/s00431-023-04923-3 (PMC10257632; doi:10.1007/s00431-023-04923-3)
Supplement: Supplementary file 1 — Supplementary file1 (PDF 166 KB) [file 431_2023_4923_MOESM1_ESM.pdf]

Supplement 1 Inclusion Flowchart

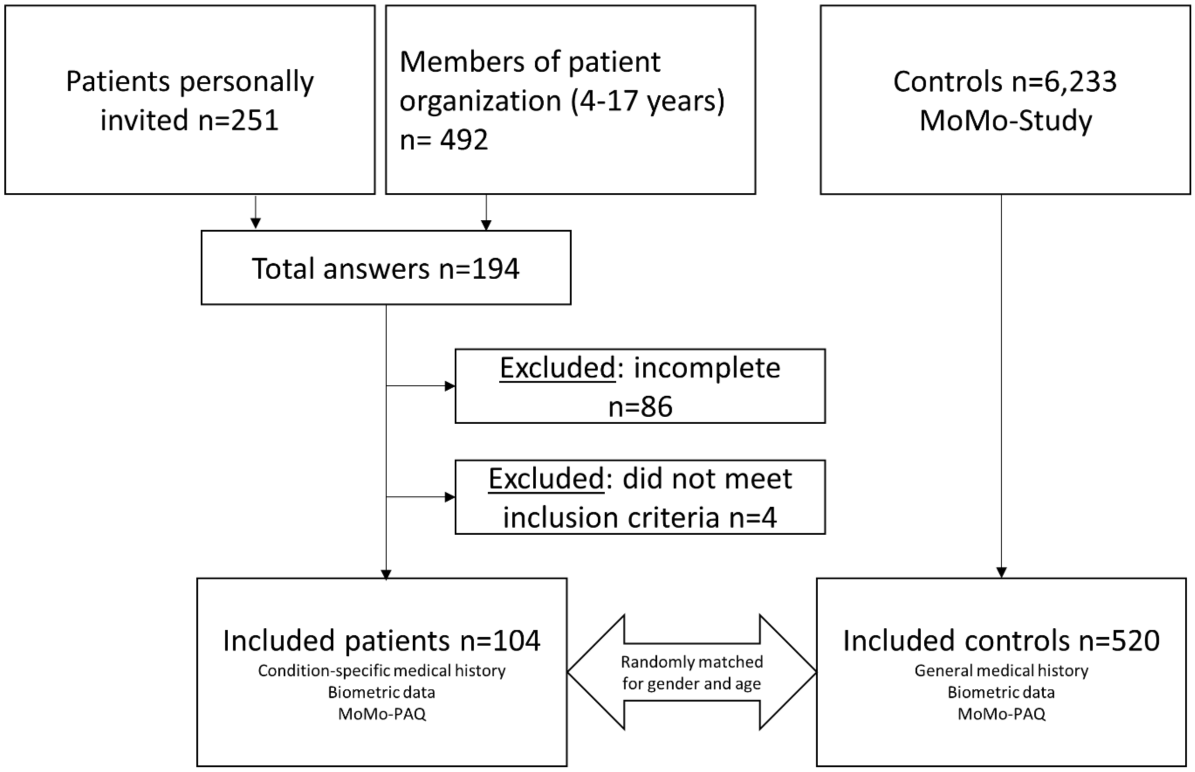

"Habitual physical activity in patients born with esophageal atresia: a multicenter cross-sectional study and comparison to a healthy reference cohort matched for gender and age."
European Journal of Pediatrics
Tatjana Tamara König*, Maria-Luisa Frankenbach, Emilio Gianicolo, Anne-Sophie Holler, Christina Oetzmann von Sochaczewski, Lucas Wessel, Anke Widenmann, Leon Klos, Simon Kolb, Jannos Siaplaouras , Claudia Niessner
* Department of Pediatric Surgery, Universitätsmedizin, Johannes Gutenberg-University Mainz, Germany, Tatjana.Koenig@unimedizin-mainz.de
