# Supplementary material for: Habitual physical activity in patients born with oesophageal atresia: a multicenter cross-sectional study and comparison to a healthy reference cohort matched for gender and age
Source: Eur J Pediatr. 2023 Mar 28;182(6):2655–63. doi: 10.1007/s00431-023-04923-3 (PMC10257632; doi:10.1007/s00431-023-04923-3)
Supplement: Supplementary file 2 — Supplementary file2 (PDF 115 KB) [file 431_2023_4923_MOESM2_ESM.pdf]

Supplement 2 Spearman rank correlation coefficient between physical activity and medical factors in patients with esophageal atresia, *significant effect ($p < 0.05$), MVPA= moderate to vigorous physical activity, EA= esophageal atresia

	**Correlation with sports index**		**Correlation with MVPA minutes**	
	**Spearman r**	**p**	**Spearman r**	**p**
Type of EA	-0.1	0.32	0.20	0.04*
Premature birth	0.08	0.43	-0.04	0.70
Primary vs. delayed anastomosis	0.07	0.50	0.57	0.57
Open vs. thoracoscopic repair	0.08	0.42	0.04	0.73
Gastrostomy	-0.60	0.12	-0.12	0.23
Fundoplication	-0.05	0.59	-0.002	0.99
Number of dilatations	-0.18	0.08	-0.20	0.84
Number of procedures under general anaesthesia	0.03	0.78	0.11	0.27
Associated malformation				
Congenital heart disease	-0.61	0.54	-0.07	0.49
Skelettal malformation	-0.81	0.07	-0.18	0.07
Urogenital malformation	-0.12	0.25	-0.20	0.04*
Anorectal malformation	-0.13	0.21	-0.24	0.01*
Current symptoms at rest	0.14	0.16	0.05	0.64
Current symptoms during exercise	-0.18	0.07	0.05	0.64

"Habitual physical activity in patients born with esophageal atresia: a multicenter cross-sectional study and comparison to a healthy reference cohort matched for gender and age."
European Journal of Pediatrics
Tatjana Tamara König*, Maria-Luisa Frankenbach, Emilio Gianicolo, Anne-Sophie Holler, Christina Oetzmann von Sochaczewski, Lucas Wessel, Anke Widenmann, Leon Klos, Simon Kolb, Jannos Siaplaouras , Claudia Niessner
* Department of Pediatric Surgery, Universitätsmedizin, Johannes Gutenberg-University Mainz, Germany, Tatjana.Koenig@unimedizin-mainz.de
