# Supplementary material for: Habitual physical activity in patients born with oesophageal atresia: a multicenter cross-sectional study and comparison to a healthy reference cohort matched for gender and age
Source: Eur J Pediatr. 2023 Mar 28;182(6):2655–63. doi: 10.1007/s00431-023-04923-3 (PMC10257632; doi:10.1007/s00431-023-04923-3)
Supplement: Supplementary file 3 — Supplementary file3 (PDF 124 KB) [file 431_2023_4923_MOESM3_ESM.pdf]

Supplement 3 Patients with specific recommendations for physical activity issued by their physician. MVPA= moderate to vigorous physical activity, EA=esophageal atresia, GERD= gastroesophageal reflux disease, CHD= congenital heart disease

**Sex, Age**	**Characteristics esophageal atresia**	**Co-morbidity**	**Symptoms**	**Sports restrictions**	**Sports index [min]**	**MVPA minutes**
Male, 7	Gross Type C, secondary anastomosis	Premature birth, skelettal malformation, anorectal malformation, underweight	GERD at rest, respiratory symptoms at rest and during exercise (even low intensity)	Resistance training or riding bikes only, no grading school sports	0	150
Male, 9	Gross Type C, primary anastomosis	Premature birth, CHD (current treatment), underweight	GERD at rest, early exhaustion, low exercise capacity	No somersault (on the ground), activity restriction during school sports	110	373
Female, 14	Gross Type C, primary anastomosis	Premature birth, urogenisal sinus, tethered chrod syndrome, enteral ostomy		No swimming in school because of enteral ostomy	216	150
Male, 12	Gross Type B	Premature birth, CHD (without therapy), congenital diaphragmatic hernia, underweight	None at rest, shortness of breath and low strength during exercise	No skiing above 2300m altitude, no restrictions during school sports	331	222
Male, 13	Gross Type C, primary anastomosis	Underweight, tracheomalazia, scapula alata	Respiratory problems and nausea during endurance training	Leisure sports: take breaks regularly, no one-sided sports, no restrictions during school sports	446	506
Male, 5	Gross Type C, primary anastomosis	Underweight, CHD (currently treated), skelettal malformation	None with regards to esophageal atresia	Leisure sports: take breaks at least every 20 minutes, no restriction during school sports	84	661
Female, 5	Gross Type C, primary anastomosis	Underweight, skelettal Malformation, anorectal malformation, urogenital malformation, tethered chrord, syrinx	None with regards to esophageal atresia	No horseback riding or jumping on the trampoline	105	105
Female, 7	Gross Type C, primary anastomosis	Premature birth, CHD (current treatment), skelettal malformation, cleft palate	GERD at rest, shortness of breath during exercise	Leisure sports: no long or highly intense physical activity	96	493
Female, 13	Gross Type C, primary anastomosis	Premature birth, scoliosis, hydrocephalus	GERD at rest	Leisure sports: no ball games, full school sports exemption	300	300
Male, 7	Gross Type C, primary anastomosis	Hemihypertrophia, hardness of hearing, branchio-oto-renal syndrome	Severe GERD at rest and during exercise	Exercise intensity depending on symptom load, school sports until symptoms occur	186	426

“Habitual physical activity in patients born with esophageal atresia: a multicenter cross-sectional study and comparison to a healthy reference cohort matched for gender and age.”
European Journal of Pediatrics
Tatjana Tamara König*, Maria-Luisa Frankenbach, Emilio Gianicolo, Anne-Sophie Holler, Christina Oetzmann von Sochaczewski, Lucas Wessel, Anke Widenmann, Leon Klos, Simon Kolb, Jannos Siaplaouras , Claudia Niessner
* Department of Pediatric Surgery, Universitätsmedizin, Johannes Gutenberg-University Mainz, Germany, Tatjana.Koenig@unimedizin-mainz.de

“Habitual physical activity in patients born with esophageal atresia: a multicenter cross-sectional study and comparison to a healthy reference cohort matched for gender and age.”
European Journal of Pediatrics
Tatjana Tamara König*, Maria-Luisa Frankenbach, Emilio Gianicolo, Anne-Sophie Holler, Christina Oetzmann von Sochaczewski, Lucas Wessel, Anke Widenmann, Leon Klos, Simon Kolb, Jannos Siaplaouras , Claudia Niessner
* Department of Pediatric Surgery, Universitätsmedizin, Johannes Gutenberg-University Mainz, Germany, Tatjana.Koenig@unimedizin-mainz.de
