# Supplementary material for: Habitual physical activity in patients born with oesophageal atresia: a multicenter cross-sectional study and comparison to a healthy reference cohort matched for gender and age
Source: Eur J Pediatr. 2023 Mar 28;182(6):2655–63. doi: 10.1007/s00431-023-04923-3 (PMC10257632; doi:10.1007/s00431-023-04923-3)
Supplement: Supplementary file 4 — Supplementary file4 (PDF 107 KB) [file 431_2023_4923_MOESM4_ESM.pdf]

Supplement 3 Spearman rank correlation coefficient between physical activity and psychosocial factors in patients with esophageal atresia, *significant effect (p< 0.05), MVPA= moderate to vigorous physical activity

	**Correlation sports index**		**Correlation MPVA minutes**	
	**Spearman r**	**p**	**Spearman r**	**p**
Father physically active	0.11	0.28	-0.08	0.40
Father member of a Sports Club	0.13	0.21	0.13	0.19
Mother physically active	-0.01	0.94	-0.3	0.74
Mother member of a Sports Club	0.19	0.06	-0.7	0.51
Siblings physically active	0.17	0.08	-0.05	0.59
Siblings member of a sports club	0.27	0.02*	0.04	0.72

“Habitual physical activity in patients born with esophageal atresia: a multicenter cross-sectional study and comparison to a healthy reference cohort matched for gender and age.”
European Journal of Pediatrics
Tatjana Tamara König*, Maria-Luisa Frankenbach, Emilio Gianicolo, Anne-Sophie Holler, Christina Oetzmann von Sochaczewski, Lucas Wessel, Anke Widenmann, Leon Klos, Simon Kolb, Jannos Siaplaouras , Claudia Niessner
* Department of Pediatric Surgery, Universitätsmedizin, Johannes Gutenberg-University Mainz, Germany, Tatjana.Koenig@unimedizin-mainz.de
