# Supplementary material for: Habitual physical activity in patients born with oesophageal atresia: a multicenter cross-sectional study and comparison to a healthy reference cohort matched for gender and age
Source: Eur J Pediatr. 2023 Mar 28;182(6):2655–63. doi: 10.1007/s00431-023-04923-3 (PMC10257632; doi:10.1007/s00431-023-04923-3)
Supplement: Supplementary file 6 — Supplementary file6 (PDF 111 KB) [file 431_2023_4923_MOESM6_ESM.pdf]

Supplement 6 Physical activity and participation in organized sports of the participants‘ families, *significant difference ($p< 0.05$, χ^2-test), a. fraction of sports club members, b. fraction of children, who had siblings

	**Patients**	**Control**	**p**
Participant member of a sports club	60 (58%)	325 (62%)	0.36
Participation in sports competitions	15 (25%, a)	131 (40%, a)	<0.01*
Father physically active	58 (56%)	252 (48%)	0.12
Unknown	5 (4.8%)	16 (3.1%)	
Father member of a sports club	32 (31%)	167 (32%)	0.67
Unknown	1 (1.0%)	18 (3.5%)	
Mother physically active	55 (53%)	305 (59%)	0.20
Unknown	2 (1.9%)	18 (3.5%)	
Mother member of a sports club	31 (30%)	166 (32%)	0.55
Unknown	0	14 (2.7%)	
Siblings physically active	57 (73%, b)	332 (73%, b)	0.94
Siblings members of a sports club	46 (59%*)	268 (59%*)	0.96
No siblings	26 (25%)	63 (12%)	< 0.01*
Unknown	0	0	

“Habitual physical activity in patients born with esophageal atresia: a multicenter cross-sectional study and comparison to a healthy reference cohort matched for gender and age.”
European Journal of Pediatrics
Tatjana Tamara König*, Maria-Luisa Frankenbach, Emilio Gianicolo, Anne-Sophie Holler, Christina Oetzmann von Sochaczewski, Lucas Wessel, Anke Widenmann, Leon Klos, Simon Kolb, Jannos Siaplaouras , Claudia Niessner
* Department of Pediatric Surgery, Universitätsmedizin, Johannes Gutenberg-University Mainz, Germany, Tatjana.Koenig@unimedizin-mainz.de
